# The role of F-actin in the transport and secretion of chromaffin granules: an historic perspective

**DOI:** 10.1007/s00424-017-2040-9

**Published:** 2017-07-20

**Authors:** Luis M. Gutiérrez, José Villanueva

**Affiliations:** Instituto de Neurociencias de Alicante, Consejo Superior de Investigaciones Científicas-Universidad Miguel Hernández, Sant Joan d’Alacant, 03550 Alicante, Spain

**Keywords:** Chromaffin granules, F-actin, Myosin II, Myosin V, Neurosecretory cells, Exocytosis

## Abstract

Actin is one of the most ubiquitous protein playing fundamental roles in a variety of cellular processes. Since early in the 1980s, it was evident that filamentous actin (F-actin) formed a peripheral cortical barrier that prevented vesicles to access secretory sites in chromaffin cells in culture. Later, around 2000, it was described that the F-actin structure accomplishes a dual role serving both vesicle transport and retentive purposes and undergoing dynamic transient changes during cell stimulation. The complex role of the F-actin cytoskeleton in neuroendocrine secretion was further evidenced when it has been proved to participate in the scaffold structure holding together the secretory machinery at active sites and participate in the generation of mechanical forces that drive the opening of the fusion pore, during the first decade of the present century. The complex vision of the multiple roles of F-actin in secretion we have acquired to date comes largely from studies performed on traditional 2D cultures of primary cells; however, recent evidences suggest that these may not accurately mimic the 3D in vivo environment, and thus, more work is now needed on adrenomedullary cells kept in a more “native” configuration to fully understand the role of F-actin in regulating chromaffin granule transport and secretion under physiological conditions.

The filamentous cytoskeletal protein, actin, is the most abundant protein in a multitude of eukaryotic cells, playing a fundamental role in the support of the cell shape and structure, as well as in a variety of cellular processes. Among these processes, it is well stablished that F-actin is a key protein participating not only in the transport of organelles containing active substances but also in the very central events leading to the release of neurotransmitters and hormones by exocytosis [21, 31, 34, 44, 59].

Initially, it was postulated that F-actin, forming a dense meshwork in the cortical area of neuroendocrine cells, was a retentive system, preventing organelles from accessing the plasma membrane [3, 11, 58]. In the late years, however, it has been shown that the plasticity of the F-actin cytoskeleton allowed for a much more complex function, supporting simultaneously multiple roles as a retentive, as well as transportation system, and structural scaffold for the exocytotic secretory machinery. In the present work, we will review this evolution of our concept of the role of F-actin cytoskeleton in exocytosis in the model of neuroendocrine chromaffin cells.

## The pioneer works, the establishment of the “barrier” concept

**Fig. 1:**
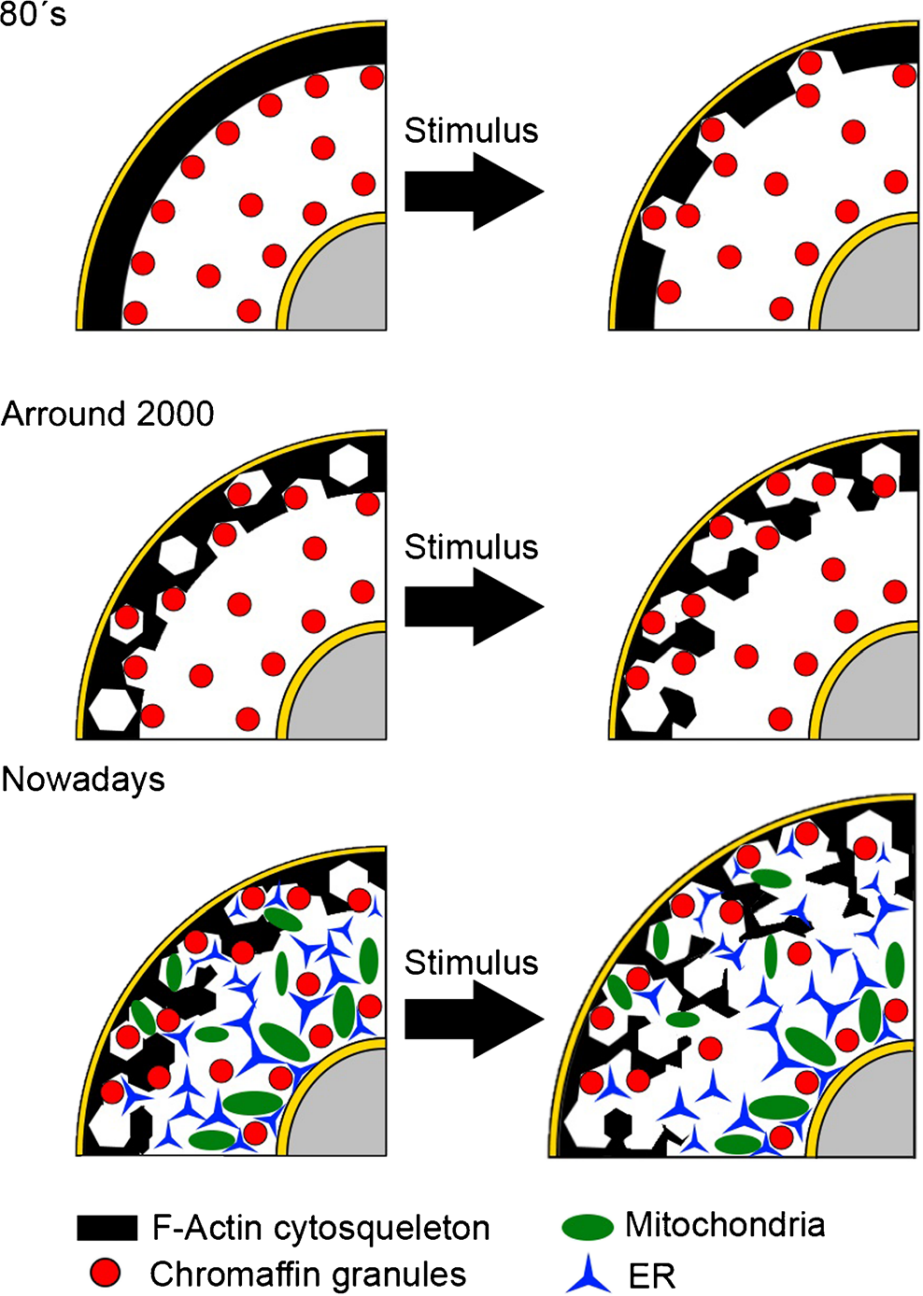
Historic evolution of the role of F-actin cortex in the secretory process of chromaffin cells. The first works in the 1980s conceived the F-actin cortex as a retentive barrier preventing the access of chromaffin granules to the secretory sites. In this context, cell stimulation resulted in the opening of the spaces that allow the access of vesicles to the submembranal zone. Around 2000, the concept evolved to incorporate a dual role as a retentive system and also transport was facilitated during exocytosis by dynamic changes in the F-actin cortical structures. Today, we accept that the cortical F-actin structure is involved in the ubication and transport of other organelles such mitochondria, and that during stimulation, multiple dynamic changes cooperate to produce the coordinated transport of vesicles to active sites (F-actin dynamic cages and the “casting system”)

At the beginning of the 1980s, with the generalization in the use of immunofluorescence techniques, several works from different laboratories evidenced that the cytoskeletal protein actin, in its filamentous form (F-actin), localized in the peripheral cortex of cultured bovine chromaffin cells forming a continuous ring [4, 29]. These studies were the base embodying the notion of the “barrier” concept for the F-actin cytoskeleton opposing the free access of secretory vesicles to the plasma membrane releasing sites [3, 8, 60] (See Fig. 1). Years later, the use of the quick-freeze, deep-etch technique in electronic microscopy [36] revealed the details of this dense meshwork of filaments and showed that some areas of the cortex presented discontinuities in this “barrier”, as the F-actin fibers where sparse or ran perpendicular to the plasma membrane.

The “barrier” idea was further supported when stimulation of the cells with secretagogues induced the reorganization of a spectrin-like protein, fodrin, forming membrane patches that co-localize with disruption in the cortical F-actin structure [45]. Similarly, cell stimulation with secretagogues induced the fragmentation of the F-actin cortex [11], a process that was associated with the activity of scinderin, a calcium-dependent protein thought to enhance the granules to be release during fast exocytosis [64]. Alternatively, calcium could affect the crosslinking properties of F-actin by protein kinase C (PKC) phosphorylation of myristoylated alanine-rich C kinase substrate (MARKS), causing the partial depolymerisation [50, 57].

In some of these early studies, it was evidenced that the role of F-actin was far more complex than a simple retentive system, as for example, the treatment of PC12 cells with Botulinum toxin C2, an ADP-ribosylating agent inducing F-actin depolymerisation, has a complex effect favoring secretion at low concentrations and inhibiting the secretory process at higher doses [32]. In addition, discrepancies among different groups stressed the complexity of the F-actin role during exocytosis since there were reports of apparent secretory enhancement and inhibition with the use of substances stabilizing (jasplakinolide or phallotoxins) or disrupting F-actin polymerization (cytochalasins, latrunculins, or clostridial C2 toxin) [15, 17, 32].

## A “dynamic” view, the dual role as a transport and retentive system

With the development of the total internal reflection fluorescence microscopy (TIRFM) and the end of the 1990s, the “barrier” concept was subjected to an important revision. This technique allowed the visualization of fluorescent-labelled granules located within 300 nm of the cell limits and therefore immersed in the F-actin meshwork [28, 41]. These studies showed that F-actin trails guided the motion of the vesicles within the F-actin-rich cortical region even though they experienced increased restrictions as they reach the immediate proximity of the plasma membrane [25]. Indeed, dynamic confocal microscopy confirmed later that chromaffin vesicles were transported from the inner regions adjacent to the cell nucleus using both microtubules and F-actin fibers and that once the reach the cell cortex, the motion was dependent mostly of the F-actin-myosin system [39, 51].

How can F-actin cortex possibly act as a retentive and also as a transport system? The first vision of the F-actin dynamic changes encompassing secretion was provided by transmitted light scanning combined with fluorescence microscopy using high numerical aperture objectives [19]. In this study, it was revealed that during stimulation, the F-actin structure parallel to the plasma membrane changes transiently to form open spaces and transport tubes allowing access of secretory granules to the secretory sites. These complex reorganizations of F-actin are the basis to understand how the cytoskeletal network can switch from a “retentive” to a “transport” system in a time window of seconds to restore the original disposition 40–50 s after the initiation of the stimulus and represented a dynamic alternative to the previous ideas based on the simple fragmentation of the F-actin “barrier” (Fig. 1, middle row). In this study, it was observed also the formation of subplasmalemmal spaces devoid of F-actin during prolonged (5 min) stimulation [19], also consistent with previous electron microscopy studies [36].

## Dynamic changes required molecular motors

F-actin changes associated with many cellular processes are driven by molecular motors of the myosin family. Myosin II, undergoing ATP-dependent conformational changes to regulate F-actin dynamics, was proved to be present in the cortical area of chromaffin cells in 1984 [60] and to be regulated by calcium-dependent phosphorylation during the secretory cycle [9, 22, 23]. The importance of the activity of such a motor of myosin II was later demonstrated when the inhibition of the myosin light chain kinase partially affected secretion [26, 46].

In addition to myosin II, myosin V was also abundant in the cytosol of chromaffin cells [38, 48] and was found to associate with chromaffin granules [49]. Importantly, it was proven that myosin V regulates the association of the vesicles with the cortical cytoskeleton in PC12 cells [51]. Myosin Va seems to be essential for the motion of the vesicles in the subplasmalemmal area, an activity that is mediated by the small GTPase Rab 27 and the MyRIP protein [12].

More recently, it was shown that myosin VI was able to recruit vesicles to the cortical zone in a calcium-dependent process; this depends on the small insert isoform located in the cargo domain of myosin VI [55]. This mean that conventional myosins V and VI could be playing similar roles operating in concert to ensure the supply of vesicles during the exhaustion of vesicular pools driven by prolonged stimulation.

Taken together, an actual and integrated view of chromaffin granule transport associated with F-actin depicts two different mechanisms acting in concert, a processive motion of vesicles along F-actin trails using conventional myosins V and VI and a non-processive transport of the granules entrapped in F-actin cages and controlled by the activity of myosin II.

## Not only transport! Actomyosin role in the fusion event

The study of the fusion kinetics with amperometry at the single vesicle fusion level [67] has been instrumental in showing that myosin motors influence not only vesicle transport but the very final events of membrane fusion. In 2004, Neco and colleagues expressed a non-phosphorylatable form of myosin II in chromaffin cells and found that F-actin and myosin II influence the kinetics of catecholamine release through the fusion pore [40], specifically prolonging the open time as was described later by using the patch amperometry technique [37]. Similarly, the pharmacological inhibition of F-actin polymerization and myosin II activity slowed release kinetics without affecting quantal size [6].

Recent works suggest that F-actin-myosin II may exert a tensional force facilitating neurotransmitter release by acting either at the plasma membrane-vesicle interface [16] or at the level of a granule coat [35]. In chromaffin cell, it is likely that actomyosin forces influence the plasma membrane tension to drive relaxation after F-actin stabilization [62], decrease membrane tension after myosin inhibition [7], and synchronize vesicle transport during stimulation [42]. In any case, the exquisite control of the cytoskeletal actomyosin appears to regulate even the mode of fusion promoting the “kiss-and-run” partial release at low-frequency stimulations and enhancing the full collapse of the granules at higher frequencies by destabilizing the F-actin structures [13]. Similarly, experiments conducted in PC 12 cells and the expression of vesicular “cargo” proteins with variable size and, therefore, diffusion rates through the fusion pore confirmed these results [1].

## The interphase between the plasma membrane and the cytoskeleton

There is no doubt that the F-actin cortical structure is a central piece of the organization of the secretory machinery, but there are also many evidences that the polymerization of F-actin “de novo” is also playing an important role.

In chromaffin cells, a molecular cascade involving small GTPases, such as Cdc42, trigers the formation of actin filaments in the submembranal area [15], and this increases the secretory activity. This F-actin recruitment seems to be mediated throught N-WASP and the Arp2/3 complex, two factors governing actin nucleation during propulsion of secretory vesicles [54]. These initial studies have been supported by a recent work probing that glycerophospholipid phosphatidylinositol 4,5-bisphosphate (PtdIns(4,5)P2) coordinates the translocation of secretory vesicles to their docking sites on the plasma membrane in a Cdc42-dependent manner [65, 66]. PtdIns(4,5)P2 forms clusters that, in addition to nucleating the formation of F-actin, also interact with SNARE proteins [2] and act as a “beacon” for vesicle guidance to active sites. The ability of F-actin to influence the modality and localization of the molecular machinery of exocytosis has recently been evidenced by the demonstration that PC12 cells emit filopodial extensions in response to secretagogue stimulation, an effect that is driven by F-actin trails that are capable of guiding secretory vesicles to newly uncovered secretory sites [43].

## F-actin involvement in the arrangement of secretory sites

Before the 1980s, it was well established that in neurons, exocytosis takes place in specialized spatial areas of the synaptic terminals called “active zones” [10, 27]. Later on, in a seminal paper, Sankaranayanan et al. showed that cortical F-actin plays a scaffold function avoiding diffusion of the molecular players of neurosecretion [52]. Therefore, a key question was if there are similar structures in neuroendocrine models, such chromaffin cells, and if F-actin plays a similar cohesive role.

Again, amperometry was seminal to show that in chromaffin cell local and restricted elevations of calcium, the so called “hot spots,” where coincident with secretory events [47]. This was the first evidence of the localized nature of the secretory response in chromaffin cells, and it was confirmed later when for most granules, calcium signals are originated within 300 nm of its location in the submembranal space [5]. Finally, using inmunolocalization techniques, it was shown that a 1/3 of SNARE microdomains co-localized with calcium channel spots and that these zones are the preferential sites for exocytosis [30]. This co-localization occurring between voltage-dependent calcium channels and secretory vesicles were first studied in early “patch clamp” studies and described in classical synapses [53].

The F-actin cytoskeleton appears to be the cohesive factor to hold these “pseudo” active sites since the expression of exogenous SNAP-25 appears to co-localize with calcium channels in the borders of F-actin cortical cytoskeletal cages [61], and this was later confirmed with the “native” proteins using immunocytochemistry [56]. Interestingly, in this study [56], it was proposed, by using mathematical models, that the organization of the secretory machinery in association with the borders of F-actin cortical structures forming cages or cavities in the subplasmalemmal space results in robust calcium confined elevations that accelerate the secretory kinetics when compared with random distributions of this machinery.

The organization of the secretory machinery has been studied in a variety of systems ranging from exocrine to neuroendocrine, and neuronal cells using atomic force and electron microscopy, leading to the description of the “porosome” as permanent cup-shaped structures associating SNARE proteins, NSF ATPase, and calcium channel subunits [24]. Among these constituents, it was described that actin was present together with other cytoskeletal proteins such as vimentin and α-fodrin.

Taking together, the emerging view suggests that the cortical F-actin cytoskeleton is an “integrative” factor associating the molecular components of the secretory machinery to configure a cytoarchitecture favoring the fine tuning of the secretory responses.

## Latest discoveries and shadows

In the later years, it has been re-inforce the role of F-actin governing the transport of the organelles, extending this control not only to chromaffin granules but also to other organelles such as mitochondria [63], playing a fundamental role in the generation of subpopulations of cortical and perinuclear organelles. In addition, “new” visions of this transport have been described to include the existence of conveyor belts to drive the displacement of entire cortical cytoplasmic regions toward the plasma membrane during cell stimulation [33]. In the proximity of the membrane, F-actin and myosin II appear to also coordinate the “casting net” system to replenish in the “docking” areas the chromaffin granules that has been released [42] (Fig. 1, lower row). Similarly, the role of F-actin during the fusion process has been sustained recently by articles enclosing new details of the molecular players. For example, the F-actin-binding protein cortactin has been implicated in the regulation of the duration of the fusion pore [20] and annexin A2 promoting F-actin-mediated bundling of the membrane is essential for the docking of the incoming granules [14].

In consequence, during the last 30 years, our vision of the role of the F-actin cytoskeleton in the secretory process in the neuroendocrine model of chromaffin cells has evolved from a simple vision of a retentive system preventing the granules to fuse in the absence of stimulation to a very complex function involving transitory dynamic changes that facilitate multiple roles as a scaffold structure supporting organelle and secretory machinery localization, coordinated organelle transport, and mechanical forces necessary for the correct opening of the fusion pore. In the context of this progress, it is important to notice, however, that most of our knowledge is based in the use of isolated and cultured cells, and recently, it has been demonstrated that the F-actin cytoskeleton of cultured chromaffin cells differs from that present in “native” cells forming part of the adrenal medulla [18]. The F-actin cytoskeleton is a complex network that extends through the whole cytosol in “native” cells, and it is destabilized in isolated cultured cells forming the “characteristic” peripheral cortical structure, and this, in consequence, changes the distribution of organelles and proteins that influence the secretory kinetics. Therefore, to fully understand the role of the F-actin cytoskeleton in “physiological” conditions, it will be necessary to develop techniques involving the use of “native cells” in a 3D in vivo like environment as found in adrenomedullary tissue.
